# Epitranscriptome profiling of spleen mRNA m^6^A methylation reveals pathways of host responses to malaria parasite infection

**DOI:** 10.3389/fimmu.2022.998756

**Published:** 2022-09-20

**Authors:** Luoluo Wang, Jian Wu, Runzhou Liu, Wenjun Chen, Zhichang Pang, Fan Zhou, Lu Xia, Jia Huang, Tao Pan, Xin-zhuan Su, Xiaoyun Wang

**Affiliations:** ^1^ School of Life Sciences, South China Normal University, Guangzhou, China; ^2^ Laboratory of Malaria and Vector Research, National Institute of Allergy and Infectious Diseases, National Institutes of Health, Rockville, MD, United States; ^3^ Department of Molecular Genetics and Cell Biology, The University of Chicago, Chicago, IL, United States; ^4^ Center for Medical Genetics, School of Life Sciences, Central South University, Changsha, China; ^5^ Department of Biochemistry and Molecular Biology, The University of Chicago, Chicago, IL, United States

**Keywords:** RNA epitranscriptome, *N^6^*-methyladenosine, malaria infection, immune response, *plasmodium yoelii*

## Abstract

*N^6^*-Methyladenosine (m^6^A), the most abundant mammalian mRNA modification, has been reported to modulate various viral infections. Although it has been confirmed that RNA modifications can also modulate the replication and development of different parasites, the role of the RNA epitranscriptome in the regulation of host response post parasite infection remains to be elucidated. Here we report host spleen m^6^A epitranscriptome landscapes induced by different strains of the malaria parasite *Plasmodium yoelii*. We found that malaria parasite infection dramatically changes host spleen m^6^A mRNA modification and gene expression. Additionally, malaria parasite infection reprograms host immune response pathways by regulating the m^6^A modification enzymes. Collectively, our study is the first characterization of host spleen m^6^A methylome triggered by malaria parasite infections, and our data identify m^6^A modifications as significant transcriptome-wide marks during host-parasite interactions. We demonstrate that host mRNA methylation machinery can sense and respond to malaria parasite infections, and provide new insights into epitranscriptomic mechanisms underlying parasite-induced pathogenesis.

## Introduction

*N^6^*-Methyladenosine (m^6^A), the most pervasive mRNA modification in mammals, can affect all aspects of mRNA biology, including RNA stability, splicing, decay, and translation (1, 2). The biological functions of m^6^A have been associated with stem cell differentiation, immune response, cancer, and others (3). As the most abundant mRNA modification in mammals, m^6^A epitranscriptomic mapping and biology have been extensively studied in the past few years (4). The epitranscriptome of m^6^A in host cells could be altered after pathogen infections, and there are growing pieces of evidence showing that m^6^A modifications play important roles in various pathogen infections including host responses to infections of SARS-CoV-2 (5), HIV (6), flavivirus (7), influenza A virus (8), hepatitis B virus (9), human metapneumovirus (10), adenovirus (11), enterovirus 71 (12). The expression of m^6^A-modified genes in host cells could be altered after virus infections. Thus, m^6^A is now considered one of the predominant epitranscriptome marks in host-pathogen interactions (13). With the advance in sequencing technologies, transcriptome-wide mappings of m^6^A and other RNA modifications after infections of pathogens have been reported, laying the foundation for a better understanding of m^6^A functions and corresponding mechanisms.

Malaria is a deadly mosquito-borne disease affecting hundreds of millions of people every year and remains a significant global health challenge (14, 15). The parasites mostly reside in red blood cells (RBCs), hiding from direct contact or recognition by host immune cells. In recent years, RNA epitranscriptome has been recognized as a new posttranscriptional modulator of gene expression during malaria parasite development, rendering RNA epigenetic marks as novel vaccine candidates (16–19). For example, m^6^A modification was associated with *P. falciparum* stage-specific fine-tuning of the transcriptional cascade and could shape the parasite transcriptome profile during blood-stage development (16). More importantly, a recent study showed that NOP2/Sun RNA Methyltransferase 2 (NSUN2)-mediated mRNA m^5^C modifications could regulate mRNA transcript stability and sexual differentiation in *Plasmodium yoelii* and *Plasmodium falciparum* (20). Additionally, DNMT2-mediated tRNA cytosine methylation in *P. falciparum* is a key regulator for the response to drug treatment and sexual commitment (21). In addition to *Plasmodium*, m^6^A enrichment was reported in 342 transcripts of *Trypanosoma brucei* including transcripts encoding variant surface glycoproteins that are essential for the survival of the parasites (22). In *Toxoplasma gondii*, m^6^A has been reported as a critical mRNA modification widespread across multiple stages of the parasite’s life cycle and is essential for parasite viability and development (23, 24). Emerging evidence has provided new insights into the roles of RNA epitranscriptome in parasite biology and the treatment of the associated diseases (25).

Although the role of m^6^A modification in the development of malaria and other parasites has been demonstrated, the host m^6^A epitranscriptomic modifications and the functional consequences post malaria parasite infection remain elusive to date. In this study, we investigate genome-wide host m^6^A RNA modifications after malaria parasite infections and show that host RNA m^6^A machinery can sense and respond to malaria parasite infections including regulation of immune response-associated pathways.

## Materials and methods

### Parasites infection and sample collection

Freshly thawed parasites (*P. y. nigeriensis* N67, *P. y. nigeriensis* N67C, *P. y. yoelii* YM, and *P. y. yoelii* 17XNL) were injected into C57BL/6 mice (aged 6-8 weeks) to initiate infections. An inoculum containing 1×10^6^ iRBCs suspended in 100 μL phosphate buffer saline from the donor mice was injected into experimental mice intraperitoneally. Four days later, the spleens and red blood cells (with malaria parasites inside) from infected or uninfected mice were rapidly separated and freshly frozen on dry ice and stored at –80°C. All animal procedures in this study were performed following the protocol approved (approval number LMVR11E) by the Institutional Animal Care and Use Committee at the National Institute of Allergy and Infectious Diseases following the guidelines of the Public Health Service Policy on Humane Care and Use of Laboratory Animals and AAALAC.

### RNA isolation and purification

Total RNA was isolated from the mice spleens and red blood cells using TRIzol (Ambion). Total RNA concentration and purity were quantified using NanoDrop 2000 Spectrophotometer (Thermo Scientific). All RNA samples were kept at –80°C until used. To obtain mRNA from mouse spleens, total RNA was purified using PolyATtract^®^ mRNA Isolation System IV (PRZ5310, Promega) followed by the RiboMinus transcriptome isolation kit for human/mouse (K155002, Invitrogen). To obtain mRNA from the malaria parasites, total RNA was purified using PolyATtract^®^ mRNA Isolation System IV (PRZ5310, Promega) followed by the RiboMinus™ Eukaryote Kit (A1083708, Invitrogen).

### LC-MS/MS quantification of m^6^A mRNA modification

The purified mRNAs (150 ng) were digested with 2 U nuclease P1 (N8630, Sigma) in 30 μL of buffer containing 25 mM NaCl and 2.5 mM ZnCl_2_ for 2 h at 37°C, followed by the addition of 2 μL FastAP Thermosensitive Alkaline Phosphatase (EF0651, Thermo Scientific) and incubation at 37°C for 4 h. The samples were then filtered through a 0.22 μm PVDF filter (Millipore) and transferred into mass spectrometry tubes. Ten μL of each sample was injected into a C18 reverse phase column coupled online to Agilent 6460 LC-MS/MS spectrometer in positive electrospray ionization mode. The nucleosides were quantified by using retention time and the nucleoside to base ion mass transitions (268-to-136 for A; 282-to-150 for m^6^A). Quantification was performed by comparing with the standard curve obtained from pure nucleoside standards running with the same batch of samples. The m^6^A/A ratio was calculated based on the calibrated concentrations.

### MeRIP-seq/m^6^A-seq

MeRIP-seq experiments were carried out according to procedures in our previous report (26). Briefly, poly(A)-selected and ribodepletion-treated mRNA samples (1.5 μg) were used for RNA fragmentation followed by immunoprecipitation with EpiMark^®^ N6-Methyladenosine Enrichment Kit (E1610S, New England Biolabs). RNAs were eluted from protein G magnetic beads in 100 μL of Buffer RLT (79216, Qiagen) followed by precipitation. The RNAs were dissolved in 12 μL RNase-free water. Input RNAs and immunoprecipitated RNAs were used for library preparation using the TruSeq Stranded mRNA kit (RS-122-2101, Illumina) according to the manufacturer’s instructions. The concentration and quality of libraries were measured using the Agilent 2100 bioanalyzer. RNA sequencing was carried out at the University of Chicago Genomics Facility on an Illumina HiSeq2500 platform that generates 100-bp paired-end reads.

### Analysis of high-throughput sequencing data

#### General processing

After removing adapters and low-quality bases using Cutadapt (v1.15), the Fastq files were aligned to the reference genome (mm10 and VSV) using Hisat2 (v2.1.0) (27). Reads mapped to tRNA and rRNA were removed and each sample obtained ~30 million useful reads for the following analysis.

#### RNA-seq and gene expression analysis

Stringtie (v1.3.3b) (28) was used to calculate the FPKM of each gene to represent their mRNA expression level. The differentially expressed genes were identified by a negative binomial model using the DEseq2 package (29), combining information from all replicates. Significantly differentially expressed genes must meet all the following criteria: q-value ≤ 0.01, log2 (fold-change) ≥ 1 or ≤ -1. KEGG enrichment analyses of differential genes were conducted by R package clusterProfiler (v3.8.1) (30).

#### m^6^A-seq analysis

The m^6^A peak calling method was modified from published report (31). In brief, the longest isoform of each gene was scanned using a 100-bp sliding window with 10-bp steps. We excluded windows with read counts less than 1/20 of the top window in both the input and m^6^A-IP sample to reduce bias from potentially inaccurate gene structure annotation and the arbitrary use of the longest isoform. The read counts in each window were normalized by the median count of all windows of that gene. The differential windows between the input and IP samples were identified with a negative binomial model by using the edgeR package (32), combing information from all replicates. A positive window was called if the false discovery rate (FDR) < 0.01 and log2 (enrichment score) ≥ 1, and overlapped positive windows were merged. The following four numbers were calculated to obtain the enrichment score of each peak (or window): reads count of the IP sample in the current peak/window (a); median reads count of the IP sample in all 100-bp windows on the current mRNA (b); reads count of the input sample in the current peak/window (c); and median reads count of the input sample in all 100-bp windows on the current mRNA (d). The enrichment score of each window was calculated as (a × d)/(b × c). Sequence motifs on m^6^A peaks were identified using HOMER (33).

### Real-time RT-PCR

The PrimeScript RT Reagent Kit (RR047A, Takara) was used to synthesize cDNA with 1 μg of total RNA from the mouse spleen. The cDNAs were 20-fold diluted for real-time RT-PCR reaction using Hieff qPCR SYBR Green Master Mix (11202ES08, YEASEN). The fluorescence intensity of the amplification process was monitored using CFX96 Touch™ Real-Time PCR Detection System (Bio-Rad). Primers used for RT-qPCR are listed in **Table 1**. Actin was used as the internal control, and the relative expression levels of target mRNAs were calculated using the 2^−ΔΔCt^ method.

**Table 1:** Primers used for RT-qPCR in this study.

Gene	Forward primer	Reverse primer
Irf1	ATGCCAATCACTCGAATGCG	TTGTATCGGCCTGTGTGAATG
Irf7	GAGACTGGCTATTGGGGGAG	GACCGAAATGCTTCCAGGG
Ifit2	AGTACAACGAGTAAGGAGTCACT	AGGCCAGTATGTTGCACATGG
Ifngr2	TCCTCGCCAGACTCGTTTTC	GTCTTGGGTCATTGCTGGAAG
Ifnar2	CTTCGTGTTTGGTAGTGATGGT	GGGGATGATTTCCAGCCGA
Actin	GGCTGTATTCCCCTCCATCG	CCAGTTGGTAACAATGCCATGT

### Western blotting

Protein samples were extracted using RIPA buffer (Thermo Fisher Scientific) with freshly added 1 mM PMSF. The lysate was supplemented with NuPage Sample Buffer (Thermo Fisher no. NP0008) and denatured at 75°C for 10 min. The samples were subjected to polyacrylamide gel electrophoresis, transferred onto a polyvinylidene difluoride membrane, and then blocked for 1 h at room temperature in 1% milk with 0.1% Tween-20 in PBS. Primary antibodies included rabbit anti-METTL14 antibody (HPA038002, Sigma, 1: 3,000), rabbit anti-METTL3 antibody (ab195352, Abcam, 1: 1,000), rabbit anti-ALKBH5 antibody (HPA007196, Sigma, 1: 1,000), mouse anti-FTO antibody (ab92821, Abcam, 1: 1,000), mouse anti-beta actin antibody (ab6276, Abcam, 1: 5,000). The membranes were probed with primary antibodies for 2 h at RT, washed 3X with blocking buffer, and with secondary antibodies conjugated to HRP (1:10,000). The HRP signal was developed using Amersham™ ECL™ Western Blotting Detection Reagents (GE Healthcare). The intensities of the protein bands were quantified using the Image Lab Software.

### Statistical analysis

Data are presented as mean ± standard deviation (SD). Asterisks represent significant differences between samples, as determined by the Mann-Whitney test or Student’s t test (P < 0.05).

## Results and discussion

### Malaria parasite infections in mice alter host spleen mRNA m^6^A methylation levels

**Figure 1:**
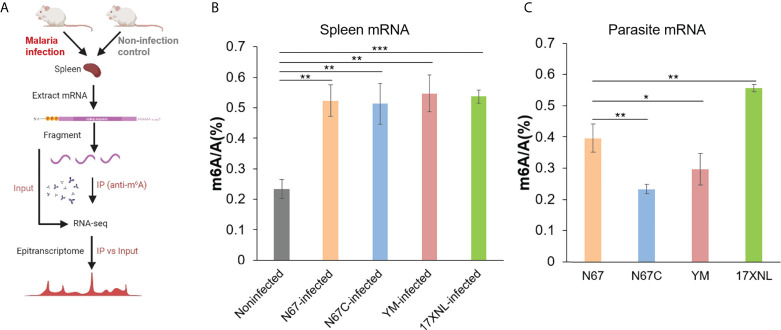
Malaria parasite infections in mice alter host spleen mRNA m^6^A methylation levels. **(A)** Schematic of the experimental design and MeRIP-seq protocol used in this study to identify differential m^6^A methylation profiles in mice spleens following infection with Plasmodium yoelii parasites. **(B)** LC-MS/MS quantification of m^6^A level in poly(A)-selected and RiboMinus-treated mRNAs isolated from the spleens of noninfected, P. y. nigeriensis N67-, P. y. nigeriensis N67C-, P. y. yoelii YM-, or P. y. yoelii 17XNL- infected mice. **(C)** LC-MS/MS quantification of m^6^A level in poly(A)-selected and RiboMinus-treated mRNAs isolated from N67, N67C, YM and 17XNL parasites. Values are the means  ± SD, n = 3. *P < 0.05; **P < 0.01; ***P < 0.001, Student’s t test.

To investigate host response to malaria parasite infection regulated by m^6^A modification, we infected mice with four *P. yoelii* strains, including N67, N67C, YM, and 17XNL in parallel with noninfected controls. These four strains of *P. yoelii* trigger dramatically different disease phenotypes in mice (14). Four days post-infection, the spleens and blood samples were collected from mice for total RNA extraction and mRNA purification. We measured total m^6^A/A ratios of mRNA using liquid chromatography-tandem mass spectrometry (LC-MS/MS), and investigated mRNA m^6^A epitranscriptome profiles using m^6^A-MeRIP sequencing and data analysis (**Figure 1A**). LC-MS/MS data showed that the average m^6^A levels in all *P. yoelii-*infected host spleen mRNAs (0.52~0.55%) were significantly higher than that in noninfected mRNA (0.23%, **Figure 1B**). In addition, m^6^A levels in four *P. yoelii* strains were diverse (**Figure 1C**), with the highest level of m^6^A modification in 17XNL (0.56%) and the lowest level in N67C (0.23%).

Although m^6^A modification has been extensively studied in mammals, the level and function of m^6^A modification in species such as parasites and other infectious pathogens are limited. Especially for the malaria parasites, the m^6^A RNA epitranscriptome was recognized as an important posttranscriptional regulator of gene expression for the parasite development very recently (16–19); however, the profiles of m^6^A modification levels in different malaria strains and spleens of infected-hosts remain unclear. Here, we showed that m^6^A modification levels in malaria parasite could be variable among strains and m^6^A modification levels in the host could be altered post malaria infection. Considering m^6^A epitranscriptome profiles induced by other pathogens in previous studies and the dramatic changes in mRNA m^6^A level post malaria parasite infection, we next performed m^6^A-MeRIP sequencing to obtain transcriptome-wide maps of m^6^A modifications. For the sequencing in this study, we focused on the spleen mRNA m^6^A profiles induced by N67 (N67Infected) and N67C (N67CInfected) in comparison with noninfected control due to their unique disease phenotypes and their highly similar genome sequences.

### Malaria parasite infections in mice alter host spleen mRNA m^6^A methylome profiles

**Figure 2:**
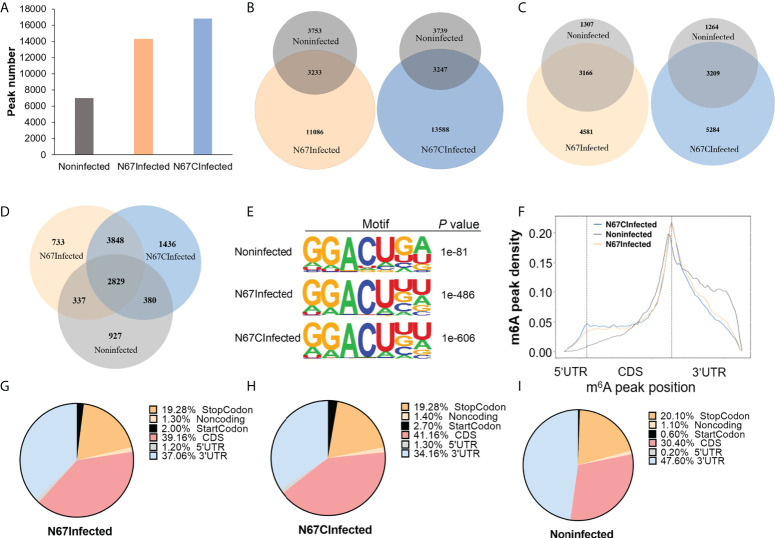
Malaria parasite infections in mice alter host spleen mRNA m^6^A methylome profiles. **(A)** m^6^A peak number identified from MeRIP in N67-, N67C-infected or noninfected spleens. **(B)** Venn diagrams showing the differences and overlaps of m^6^A peaks identified from MeRIP between N67- or N67C-infected and noninfected spleens, respectively. **(C)** Venn diagrams showing the differences and overlaps of m^6^A-containing genes between N67- or N67C-infected and noninfected spleens, respectively. **(D)** Venn diagram showing the differences and overlaps of m^6^A-containing genes among N67-, N67C-infected and noninfected spleens. **(E)** Consensus motifs and corresponding P values of m^6^A peaks identified by HOMER from m^6^A-seq analysis. **(F)** m^6^A peak distribution across the mRNA regions (5’UTR, CDS, 3’UTR). m^6^A peaks were mapped to the corresponding gene and plotted according to their position. **(G)** Pie chart showing the proportion of m^6^A peaks in different mRNA regions of N67-infected mice spleens. **(H)** Pie chart showing the proportion of m^6^A peaks in different mRNA regions of N67C-infected mice spleens. **(I)** Pie chart showing the proportion of m^6^A peaks in different mRNA regions of noninfected mice spleens.

From the m^6^A MeRIP-seq data analysis, we identified 14319, 16835, and 6986 m^6^A peaks for N67Infected, N67CInfected, and noninfected spleens, respectively (**Figure 2A**). Approximately 80% of differential peaks were induced by malaria parasite infection, and only 20% of the m^6^A peaks in the infected samples (22.58% for N67Infected and 19.29% for N67CInfected) overlapped with noninfected samples (**Figure 2B**). Correspondingly, 4581 and 5284 m^6^A peak-containing genes were mapped to mouse spleens infected with N67- and N67C-, respectively (**Figure 2C**). Among them, 3848 m^6^A peak-containing genes were shared by N67- and N67C-infected samples, and 733 and 1436 m^6^A peak-containing genes were unique for N67- and N67C-infected samples, respectively (**Figure 2D**). We next performed epitranscriptome analysis to investigate the sequence features of m^6^A modification post malaria parasite infections. The known m^6^A motif RRACH (where R represents G or A; H represents A, C, or U), particularly GGACU, was enriched in the identified peaks in the three groups of samples (**Figure 2E**). The peak distribution analysis showed that N67-/N67C- infection lead to an obvious increase in m^6^A peak density in the regions of 5’UTR and CDS, but a decrease in the 3’UTR region (**Figure 2F**). A detailed presentation of m^6^A peak distribution confirmed that malaria parasite infection induced a dramatic increase of mRNA m^6^A modification around 5’UTR, start codon, and CDS (**Figures 2G–I**). These results demonstrate that malaria parasite infections in mice can dramatically alter host spleen mRNA m^6^A methylation levels and m^6^A methylome profiles.

### Malaria parasite infection-induced m^6^A modifications impact host gene expression

Next, we analyzed overall gene expression patterns in mice spleens post malaria parasite infection using Input samples. Principal component analysis showed that both Input samples and IP samples for N67Infected (**Figure S1A**) and N67C-infected samples (**Figure S1B**) could be separated from noninfected samples. Volcano plots and heap maps showed that large members of genes were differentially expressed between N67Infected and noninfected samples (**Figure S2A, Figure S3A**) or between N67CInfected and noninfected samples (**Figure S2B, Figure S3A**). Although the gene expression patterns were similar between N67Infected and N67CInfected samples, there were still hundreds of differentially expressed genes (**Figures S2C, S3B**). Gene Ontology analysis using differentially expressed genes showed that a substantial group of pathways related to innate immune responses were enriched upon malaria infection both for N67- (**Figure S4A**) and N67C-infected (**Figure S4B**) samples, which was consistent with a previous report (14).

**Figure 3:**
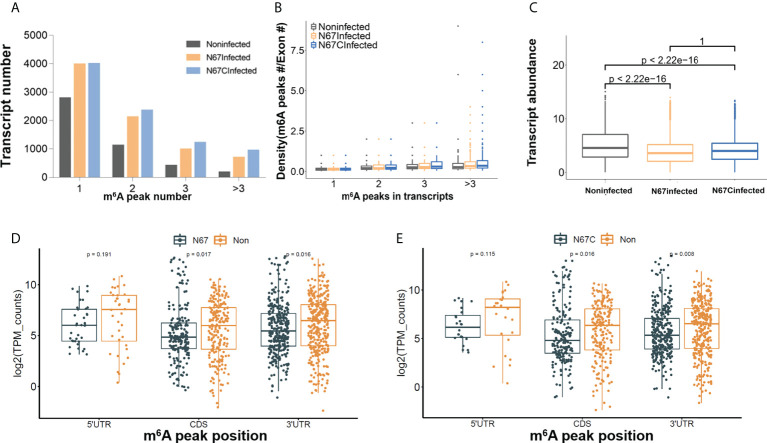
Malaria parasite infection-induced m^6^A modifications impact host gene expression patterns. **(A)** Total number of transcripts containing different m^6^A sites in N67- or N67C-infected or noninfected spleens, respectively. **(B)** Plots of m^6^A peaks within exons in N67- or N67C-infected or noninfected spleens, respectively. **(C)** Box plots showing the abundance of m^6^A-containing transcripts in N67-, N67C-infected or noninfected spleens, respectively. Transcript abundance was shown in log2 transform. **(D)** Boxplot showing expression level of genes with peaks enriched in 5’UTR, CDS and 3’UTR in N67Infected and noninfected samples. **(E)** Boxplots showing expression level of genes with peaks enriched in 5’UTR, CDS and 3’UTR in N67CInfected and noninfected samples. R package ggboxplot was used for plots and Kruskal-Wallis test was used to show the significance between groups.

To decipher the role of m^6^A modifications in the host response to malaria parasite infection, we further performed an in-depth m^6^A epitranscriptome analysis using m^6^A-IP samples. First, the transcripts with various numbers of m^6^A peaks are different in three groups. For example, among 7747, 8493, and 4473 m^6^A-containing transcripts in three groups, 3972, 3994, and 2782 transcripts have a single m^6^A peak in N67Infected, N67CInfected and noninfected samples, respectively (**Figure 3A**). We observed that the ratio for both N67-/noninfected and N67C-/noninfected was about 1.4 for one peak-containing transcript, while there were higher ratios of transcripts that were multi-methylated (e.g., 2.4 and 3.0 for N67Infected and N67CInfected samples, respectively, **Figure 3A**). Moreover, both N67Infected and N67CInfected samples had a broader distribution of m^6^A peak/exon ratio compared to the noninfected samples (**Figure 3B**). We also analyzed the expression level of transcripts with m^6^A modifications, and the results showed that the abundance of m^6^A-containing transcripts was significantly lower in N67- and N67C- infected samples than in noninfected samples (**Figure 3C**). Overall, malaria infection-induced m^6^A modifications showed a negative effect on host gene expression, considering the higher level of m^6^A modification in N67- or N67C- infected samples compared to noninfected samples. We further analyzed the expression level of genes with malaria infection-induced m^6^A peaks enriched in different mRNA regions (**Figures 3D, E**). The results showed that m^6^A peaks enriched in CDS had a negative effect on gene expression, while m^6^A peaks enriched in 3’UTR had a positive effect on gene expression. Our results suggested that malaria-induced m^6^A modification in 3’UTR may increase mRNA stability leading to higher mRNA level.

### Malaria parasite infection-induced m^6^A modifications reprogram host immune response pathways

**Figure 4:**
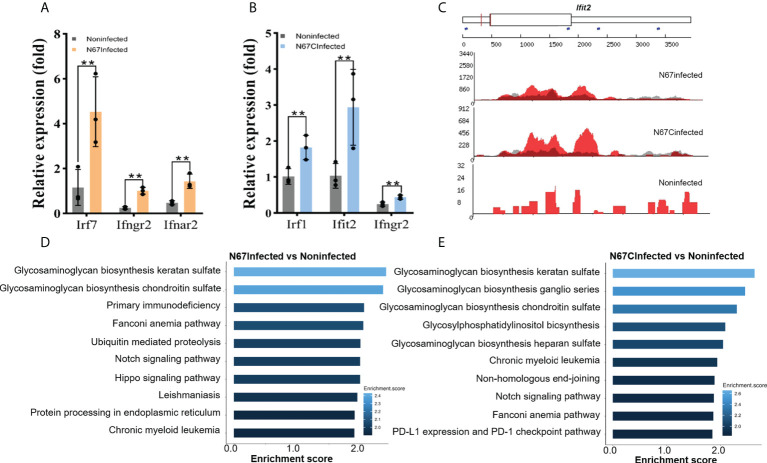
Malaria parasite infection-induced m^6^A modifications reprogram host immune response pathways. **(A)** Transcript expression of interferon stimulated genes from N67Infected and noninfected spleens, respectively. **(B)** Transcript expression of interferon stimulated genes from N67CInfected and noninfected spleens, respectively. Values are the means ± SD, *n*=3. **P < 0.01, Student’s *t* test. **(C)** Integrative genomics viewer (IGV) plots of m^6^A-IP (red) and Input (grey) showing m^6^A peaks of *Ifit2* in N67-, N67C-infected or noninfected spleens. The Y axis represents the normalized m^6^A signal along the gene. **(D)** Biological processes of differentially expressed genes with m^6^A peaks in N67-infected mice spleens compared to noninfected mice spleens. **(E)** Biological processes of differentially expressed genes with m^6^A peaks in N67C-infected mice spleens compared to noninfected mice spleens.

The overall effect of m^6^A modifications showed a negative effect on host spleen gene expression (**Figure 3C**), some immune response-related genes were still shown to be positively regulated by m^6^A modification. We have performed RT-qPCR and validated our sequencing results with specific primers targeting interferon pathway-related genes (*Irf7*, *Ifngr2*, *Ifnar2*, *Irf1*, *Ifit2*) that were previously reported in anti-parasite defense (14, 15). Those immune response-related genes gained m^6^A peaks but the expression levels were still upregulated in N67- (**Figure 4A**) or N67CInfected (**Figure 4B**) samples compared to noninfected control. For example, *Ifit2* is an interferon-stimulated gene with well-established antimicrobial activity through binding to and enhancing the translation efficiency of host mRNAs by suppressing ribosome pausing (34). Representative read coverage plots were shown for *Ifit2* (**Figure 4C**), this gene gained an obvious m^6^A peak in 3’UTR in N67CInfected samples and the expression level was dramatically up-regulated by N67C infection (**Figure 4B**), consistent with the data that m^6^A enriched in 3’UTR had a positive effect on gene expression (**Figure 3E**). A recent study showed that the translation of interferon-induced genes could be enhanced after m^6^A modification as the antiviral mechanism (35). Thus, it is conceivable that m^6^A modification on these genes may have a similar role in the immune response to malaria infections.

We further performed Gene Ontology analysis using m^6^A-marked genes. Interestingly, we found that glycosaminoglycans (GAGs) biosynthesis pathways were significantly enriched for m^6^A-modified genes in both N67Infected (**Figure 4D**) and N67CInfected samples (**Figure 4E**). GAGs are complex carbohydrates ubiquitously present on the cell surface and in the extracellular matrix, and increasing evidence indicates a key role for GAGs in the invasion of various parasitic pathogens such as *Toxoplasma*, *Plasmodium*, and *Trypanosoma* parasites (36). Therefore, our results suggest that GAGs might be the prime targets of malaria parasites through the mechanism of m^6^A modifications, which requires further investigation.

### Strain-specific immune response pathways regulated by m^6^A modifications

**Figure 5:**
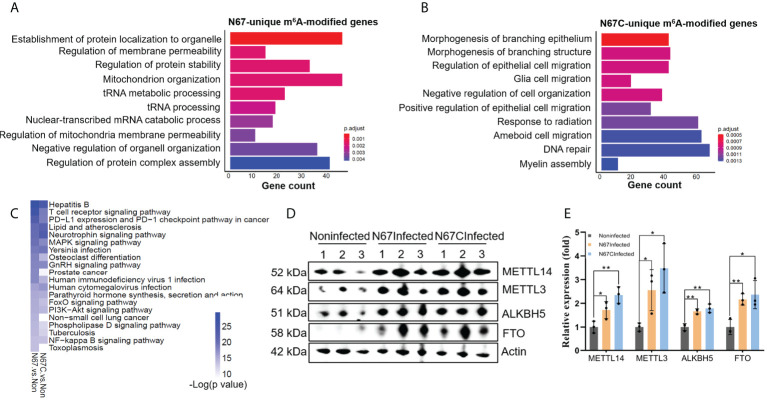
Strain-specific immune response pathways regulated by m^6^A modifications. **(A)** Biological processes of 733 N67-unique m^6^A-modified genes. Shown are the top 10 enriched pathways. **(B)** Biological processes of 1436 N67C-unique m^6^A-modified genes. Shown are the top 10 enriched pathways. **(C)** KEGG overlapping pathways of genes gained m^6^A peaks post malaria infections. Shown are the top twenty enriched pathways with low p-value for N67/noninfected and N67C/noninfected comparisons. **(D)** Western blots showing expression levels of m^6^A writer proteins METTL3, METTL14, and eraser proteins FTO, ALKBH5 in spleens of N67-, N67C-infected or noninfected mice. **(E)** Relative expression levels of m^6^A methylation machinery proteins. Signal intensity relative to β-actin was determined using Image (J). Values are the means ± SD, *n*=3. *P < 0.05, **P < 0.01, Student’s t test.

Since the two parasite strains N67 and N67C can cause different disease phenotypes though have similar genome sequences (>99% identity) (37), we tried to explore the potential differences at m^6^A epitranscriptome level. We performed Gene Ontology analysis using m^6^A-modified genes which are unique for N67 (733 genes) or N67C (1436 genes). The pathways with the highest confidence were “establishment of protein localization to organelle” and “morphogenesis of branching epithelium” for the N67 strain and N67C strain, respectively (**Figures 5A, B**). Whether m^6^A modification plays strain-specific roles in regulating different phenotypes through those strain-unique m^6^A-modified genes and associated pathways requires further investigation. We also performed KEGG analysis using malaria infection-gained m^6^A-modified genes (**Table 2**) and found that most of the enriched pathways from KEGG analysis were related to pathogen infections (**Figure 5C**), leading to the support that malaria parasite infection-induced m^6^A modifications indeed influence host immune responses.

**Table 2:** Immune response related genes gained m^6^A peaks post malaria infection.

N67-induced genes	m^6^A peak distribution	N67C-induced genes	m^6^A peak distribution
Erbb2	CDS	Fgl2	Stop Codon
Il4ra	CDS	Ythdf2	CDS
Nlrc5	CDS	Atm	Stop Codon
Cblb	3’UTR	Mafb	3’UTR
Gpr55	CDS	Fbn1	3’UTR
Bank1	CDS	Fbxw7	CDS
Lrch1	3’UTR	Slit2	CDS
Il20rb	3’UTR	Loxl3	3’UTR
Btla	CDS	Mertk	Stop Codon
Parp14	CDS	Flt3	Stop Codon
Smad7	5’UTR	Lyn	3’UTR
Cd22	CDS	Syt11	CDS
Pik3r1	5’UTR	Tsc22d3	3’UTR
Ptpn6	CDS	Cd44	Stop Codon
Adgrf5	CDS	Foxf1	3’UTR
Rc3h2	CDS	Thbs1	5’UTR
Lyn	Stop Codon	Fadd	3’UTR
Ptprc	CDS	Ubash3b	Stop Codon
Fcrl5	CDS	Pag1	CDS
Lrrc32	CDS	Htra1	Stop Codon

CDS, coding sequence.

We tried to explore the potential mechanism of m^6^A modifications in regulating host immune response to malaria infection. In mammals, dynamic m^6^A modification is maintained by the enzyme complex containing the METTL3 and METTL14 proteins, and two eraser enzymes of FTO and ALKBH5 (26). To obtain a mechanistic understanding of m^6^A changes in malaria parasite-infected mice, we measured the protein levels of m^6^A writer proteins METTL3 and METTL14, and the m^6^A eraser proteins FTO and ALKBH5 using western blot (**Figures 5D, E**, **Figure S5**). We found that both m^6^A writer proteins and m^6^A eraser proteins were highly overexpressed in the N67-/N67C-infected samples compared to that in noninfected ones. In our study, the simultaneous overexpression of the m^6^A writer complex and the erasers in N67- and N67C-infected samples suggests that mRNA m^6^A modification plays a key role in the regulation of host immune function and response to parasite infection.

In summary, we have characterized the dynamic m^6^A mRNA methylation profiles in mice infected with different *P. yoelii* strains. Our results show that m^6^A is a crucial mechanism of post-transcriptional regulation during malaria parasite infections. Malaria parasite infection dramatically changes the host m^6^A mRNA modification profile and gene expression in the spleen by regulating the m^6^A modification enzymes. Future studies include delineating specific contributions and signaling mechanisms of key molecules that regulate host m^6^A methylome during malaria parasite infections, which may help develop vaccines or drugs to combat malaria. For instance, small molecule drugs targeting enzymes of the m^6^A machinery or genes modified by malaria-induced m^6^A-machinery may activate or enhance host immune responses to control malaria infections.

## Data availability statement

The RNA-seq and m6A-seq data generated by this study have been deposited in the NCBI GEO database under the accession number GSE150546.

## Ethics statement

All animal procedures in this study were performed following the protocol approved (approval number LMVR11E) by the Institutional Animal Care and Use Committee at the National Institute of Allergy and Infectious Diseases following the guidelines of the Public Health Service Policy on Humane Care and Use of Laboratory Animals and AAALAC.

## Author contributions

X-zS and XW designed the research project. LW, JW, WC, ZP, FZ, LX, and XW performed the experiments. LW, RL, JH, and XW contributed to the data analysis. LW, JW, TP, X-zS, and XW wrote the manuscript. All authors contributed to the article and approved the submitted version.

## Funding

This work was supported by the National Natural Science Foundation of China (32070615, 81902093), the National Institutes of Health of the USA (K01 DK111764), the fellowship of the China Postdoctoral Science Foundation (2020M672676), and the Guangdong Project of Basic and Applied Basic Research (20201910240000150), and partially by the Division of Intramural Research at National Institute of Allergy and Infectious Diseases (NIAID) in National Institutes of Health (NIH), USA. XW is a recipient of Guangdong Province Universities and Colleges Pearl River Scholar Funded Scheme (2019).

## Conflict of interest

The authors declare that the research was conducted in the absence of any commercial or financial relationships that could be construed as a potential conflict of interest.

## Publisher’s note

All claims expressed in this article are solely those of the authors and do not necessarily represent those of their affiliated organizations, or those of the publisher, the editors and the reviewers. Any product that may be evaluated in this article, or claim that may be made by its manufacturer, is not guaranteed or endorsed by the publisher.
