# Supplementary material for: Epitranscriptome profiling of spleen mRNA m6A methylation reveals pathways of host responses to malaria parasite infection
Source: Front Immunol. 2022 Sep 20;13:998756. doi: 10.3389/fimmu.2022.998756 (PMC9531237; doi:10.3389/fimmu.2022.998756)
Supplement: Supplementary file 1 [file DataSheet_1.pdf]

SUPPLEMENTAL MATERIAL

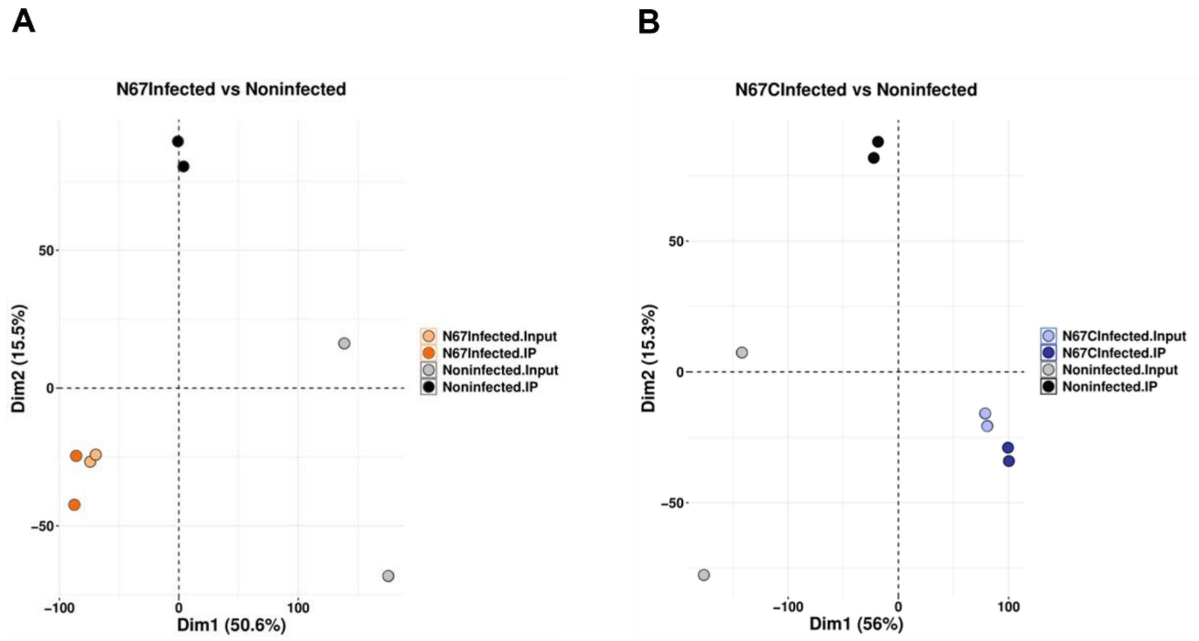

Fig. S1. Principal component analysis of input and IP samples from *Plasmodium* N67- or N67C-infected and noninfected spleens. (A) N67-infected mice spleens compared to noninfected mice spleens. (B) N67C-infected mice spleens compared to noninfected mice spleens. The eigenvalues of the top two PCs are shown; PC1 explains 50.6% and 56.0% of variance for N67- and N67C-samples, respectively; PC2 explains 15.5% and 15.3% of variance explained for N67- and N67C-samples, respectively.

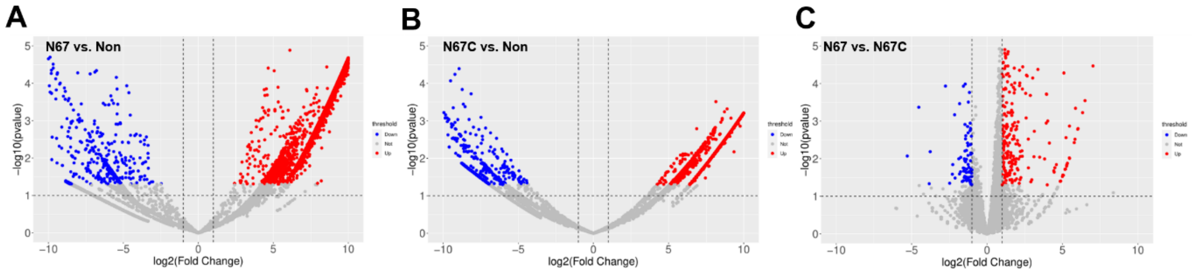

Fig. S2. Volcano plot showing up- (in red) and down-regulated (in blue) genes between different groups. (A) Comparison between N67-infected and noninfected samples. (B) Comparison between N67C-infected and noninfected samples. (C) Comparison between N67-infected and N67C-infected samples.

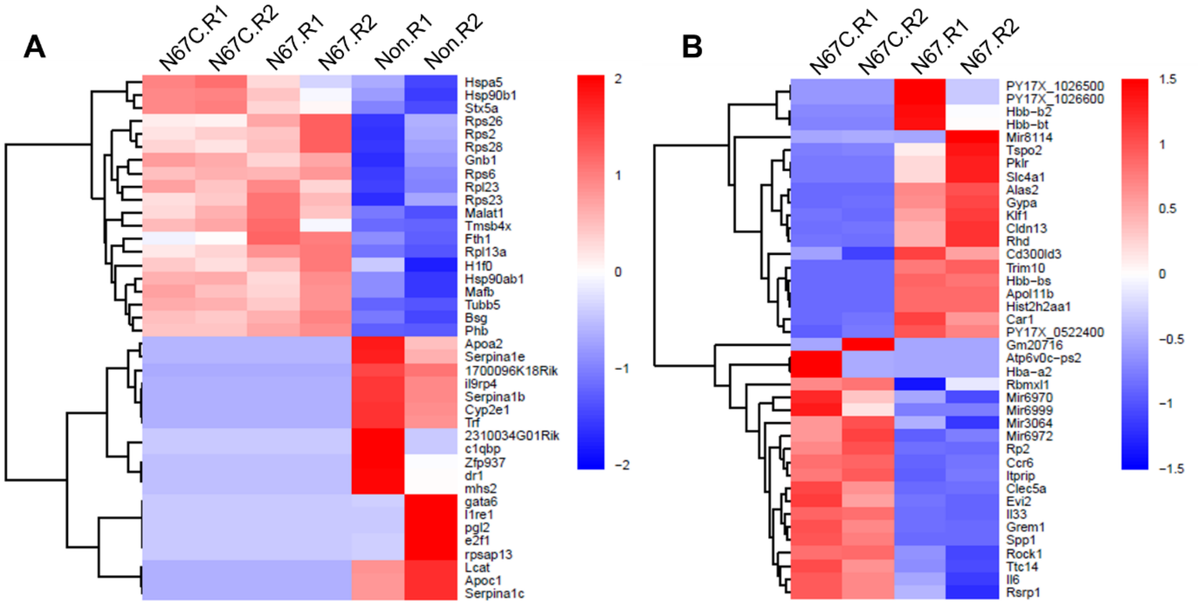

Fig. S3. Heat maps showing gene expression patterns in different groups of biological replicates. (A) The comparisons among N67-, N67C-infected and noninfected spleens. (B) The comparison between N67- and N67C-infected spleens. The expression level is indicated by red (high) and blue (low), respectively. Shown are the top 20 differentially expressed genes.

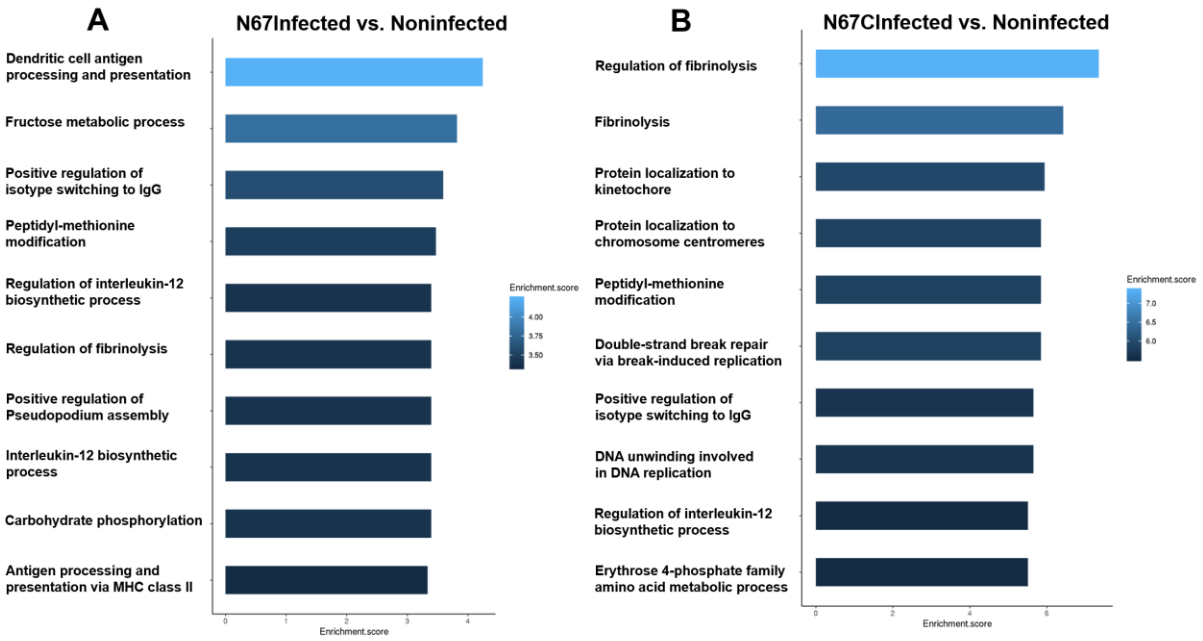

Fig. S4. Gene Ontology enrichment analysis reveals the pathways related to differentially expressed genes between groups. (A) Biological processes-related to differentially expressed genes in N67-infected mice spleens compared to noninfected mice spleens. (B) Biological processes related to differentially expressed genes in N67C-infected mice spleens compared to noninfected mice spleens. Shown are the top 10 enriched pathways.

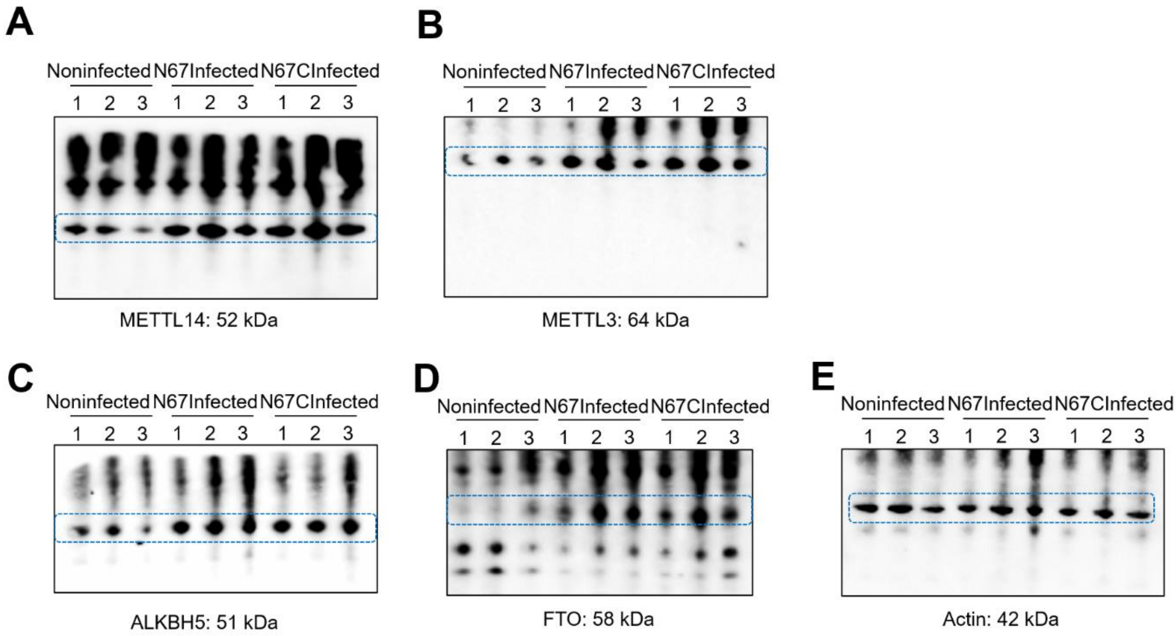

Fig. S5. Uncropped western blots of m^{6}A machinery protein levels in noninfected, N67- or N67C-infected mice spleens. (A) m^{6}A writer protein METTL14. (B) m^{6}A writer protein METTL3. (C) m^{6}A eraser protein ALKBH5. (D) m^{6}A eraser protein FTO. (E) β-actin was used as the loading control.
